# Infrasonic gliding reflects a rising magma column at Mount Etna (Italy)

**DOI:** 10.1038/s41598-022-20258-9

**Published:** 2022-10-19

**Authors:** Mariangela Sciotto, Leighton M. Watson, Andrea Cannata, Massimo Cantarero, Emanuela De Beni, Jeffrey B. Johnson

**Affiliations:** 1 Istituto Nazionale Di Geofisica E Vulcanologia, Osservatorio Etneo, Catania, Italy; 2 School of Earth and Environment, University of Canterbury, Christchurch, New Zealand; 3 Dipartimento Di Scienze Biologiche, Geologiche E Ambientali-Sezione Di Scienze Della Terra, Università Degli Studi Di Catania, Catania, Italy; 4 Department of Geosciences, Boise State University, Boise, ID USA

**Keywords:** Natural hazards, Solid Earth sciences

## Abstract

Infrasound is increasing applied as a tool to investigate magma dynamics at active volcanoes, especially at open-vent volcanoes, such as Mt. Etna (Italy), which are prodigious sources of infrasound. Harmonic infrasound signals have been used to constrain crater dimensions and track the movement of magma within the shallow plumbing system. This study interprets the remarkable systematic change in monotonic infrasound signals preceding a lava fountaining episode at Mt. Etna on 20 February 2021. We model the changing tones (0.7 to 3 Hz fundamental frequency) as a rise in the magma column from 172 ± 25 m below the crater rim to 78 ± 8 m over the course of 24 h. The infrasonic gliding disappears approximately 4 h before the onset of lava fountaining as the magma column approaches the flare of the crater and acoustic resonance is no longer supported. The featured 20 February event was just one of 52 lava fountain episodes that occurred at Mt. Etna over the course of 9 months in 2021 and was the only lava fountain episode where dramatic gliding was observed as a subsequent partial collapse of the crater prevented future resonance. The results presented here demonstrate that analysis of infrasonic gliding can be used to track the position of the magma free surface and hence may provide information on the processes taking place within the plumbing system before eruptive activity.

## Introduction

**Figure 1:**
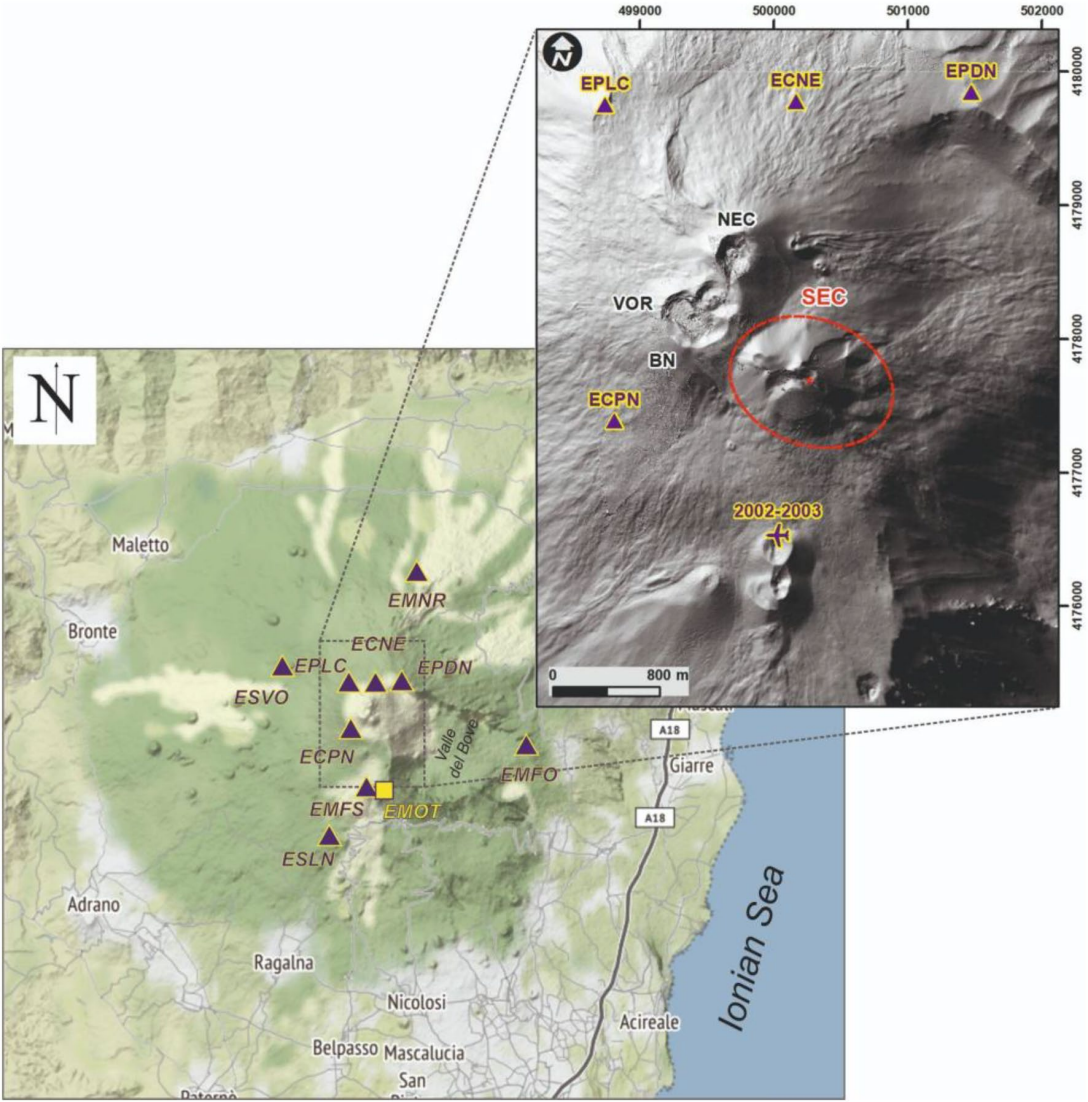
Map of Mt. Etna, showing the location of sensors belonging to the permanent infrasound network (purple triangles) and of the monitoring video camera EMOT (yellow square). Inset shows a shaded relief of the summit craters with crater names annotated (VOR, Voragine; BN, Bocca Nuova; NEC, North-East Crater; SEC, South-East Crater). The purple plane represents the takeoff point for the UAS survey. Dashed-red ellipse circumscribes the SEC, and the red circle indicates the vent where the lava fountain took place. Maps created with ArcMap 10.3 https://www.esri.com/en-us/home.

On 16 February 2021 a volcanic plume deposited ash and lapilli on Catania and other villages near Mt. Etna. The plume reached about 10 km above sea level and originated from a vent located in the area of the South-East Craters (SEC; Fig. 1) where Strombolian explosions, lava flows, and lava fountaining were also observed. The lava fountaining lasted for 3 h and reached a maximum height of 1.5 km. This eruptive episode (or paroxysm) was the first of a sequence of 52 lava fountain episodes that occurred in 2021. Such eruptive behavior is common at Etna’s SEC^1^ and is reminiscent of eruptive behavior that occurs at other volcanoes, like at Kilauea’s P’u’ ‘O’o vent in 2003^2^.

The 52 lava fountain paroxysms in the 2021 eruptive sequence followed a remarkably consistent pattern with only minor differences in event duration. Each paroxysmal episode was characterized by three main phases articulated in Alparone et al.^1^ and Andronico et al.^3^ as: (i) *resumption phase* marked by Strombolian activity resumption lasting tens of minutes to a few days, (ii) *paroxysmal phase* lasting from tens of minutes to a few hours and consisting of continuous to almost continuous lava fountains and lava overflow, and (iii) *conclusive phase* characterized by the return to low levels of eruptive phenomena until their total exhaustion. Telemetered networks operated by INGV (Fig. 1; see section Methods) recorded seismo-infrasonic waves emitted in the ground-atmosphere during eruptive activity.

The fourth lava fountain episode of the 2021 sequence (referred to as episode #4), occurring on 20 February, was observationally similar to other paroxysms in regards to the main phases characterizing the episode and their temporal succession. The lava fountaining phase lasted for three hours (approximately from 22:00 to 01:00) and produced jets with maximum height of about 1.5 km, while the eruptive plume reached around 10 km a.s.l..

This episode was unique, however, in terms of its monotonic infrasonic spectra (consisting of a single peak in the frequency domain). Strombolian activity intensified and culminated in lava fountaining after being preceded by more than 24 h of monotonic infrasound, characterized by a well-defined fundamental frequency. The monotonic infrasound began as discrete events, which became more intense and frequent and eventually merged into a continuous infrasonic tremor, which retained its monotonic signature.

Over the course of the 24 h preceding episode #4, the fundamental tone of the monotonic infrasound rose from 0.7 Hz to 3 Hz in a pattern referred to as spectral gliding. Gliding has been commonly observed in seismic tremor at a number of volcanoes (i.e. Lascar,^4^; Montserrat,^5^; Veniaminof,^6^; Redoubt,^7^), but less frequently in infrasound signals^8–10^. Our featured example at Etna is the clearest example of infrasonic spectral gliding that we have seen reported.

Seismic and acoustic gliding likely reflect changing resonance characteristics in different parts of a volcano. Several mechanisms have been proposed for seismic gliding including resonance of portions of the plumbing system, resonance of gas-filled bubbles, combination of discrete pulses producing evenly spaced harmonics, non-linear fluid flow, and/or non-linear responses to fluid flow^7,11^. Harmonic infrasound signals have been observed at open-vent volcanoes and have been attributed to Helmholtz^12,13^ or acoustic resonance of the volcanic crater^14,15^, repeating discrete pulses^16,17^, or complex source time functions^18^. Infrasonic gliding has been modeled as due to the rise or fall of magma within a resonating conduit^14,19,20^. Harmonic infrasound signals, without gliding, have previously been observed at Mt. Etna and attributed to acoustic resonance occurring between the top of the magma column and the crater outlet^13,20–22^. This study integrates infrasound along with seismic observations and visual and thermal imagery constraints to better understand the source of infrasonic gliding and to develop a quantitative model of magma column rise (position, velocity, and volume) over time. The tracking of rising magma by infrasound analysis has implications for unravelling the processes taking place within the shallow magma plumbing system and can provide higher temporal resolution than other methods such as visual overflights.

## Results

### Geophysical data

**Figure 2:**
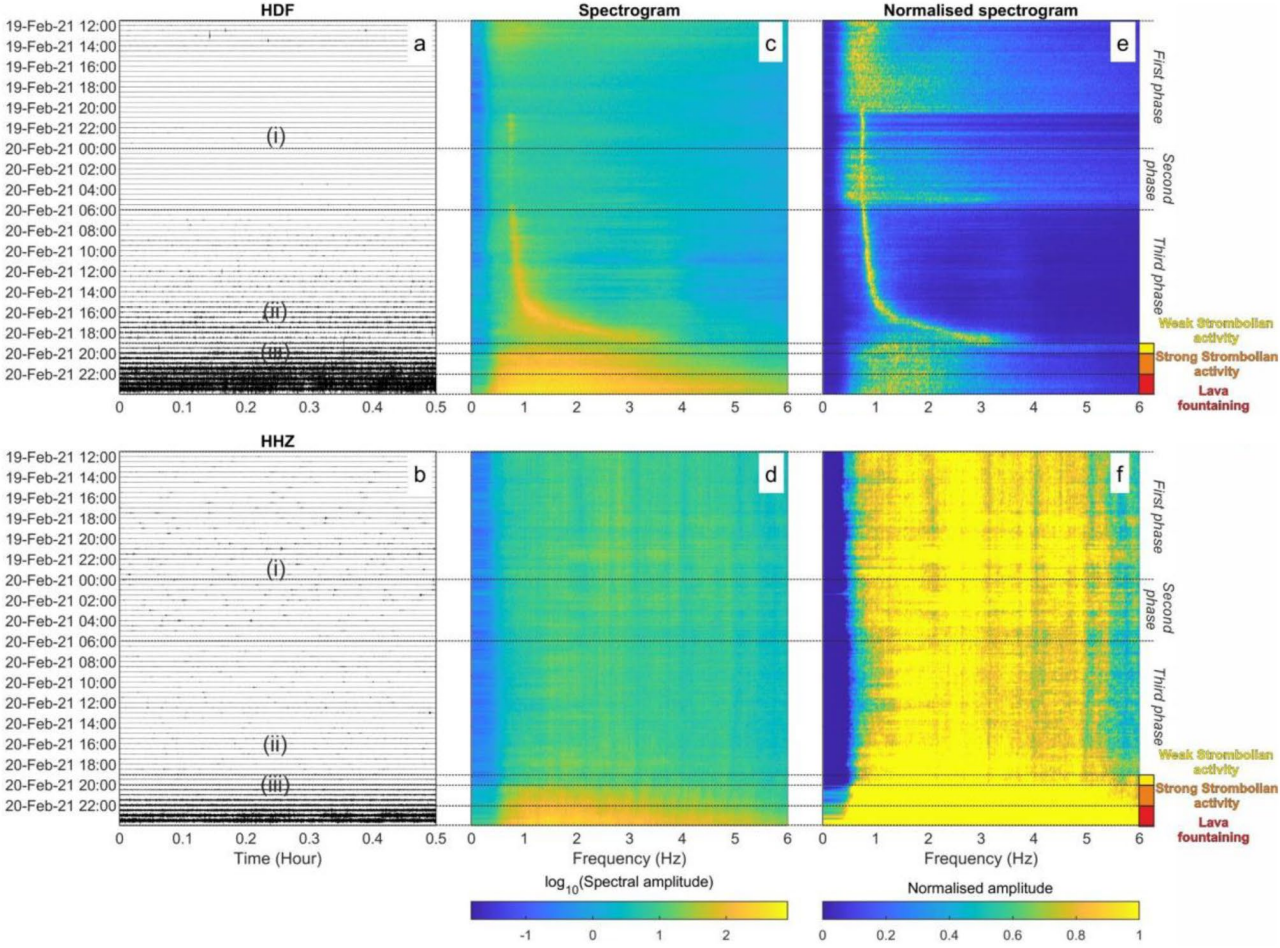
36 h of seismo-acoustic records from 19 February at 12:00 to 21 February at 00:00 recorded by ECPN. (**a**) Infrasonic signal and (**b**) vertical component of the seismic signal. (**c**, **d**) Corresponding spectrograms and (**e**, **f**) normalized spectrograms computed for hour-long intervals. The symbols (i), (ii) and (iii) in (**a**, **b**) show the time intervals when the signals shown in Fig. 3 were recorded. The black dotted lines show the different phases of the eruption, described in the text, as well as the time intervals characterised by different kinds of eruptive activity.

In order to investigate the dynamics of episode #4, we focused on 36 h of infrasonic and seismic data from 19 February at 12:00 to 21 February at 00:00 UTC (Fig. 2). This time period starts several hours before the resumption of visible volcanic activity and encompasses both the Strombolian activity and growth of the lava fountain.

### Infrasound

**Figure 3:**
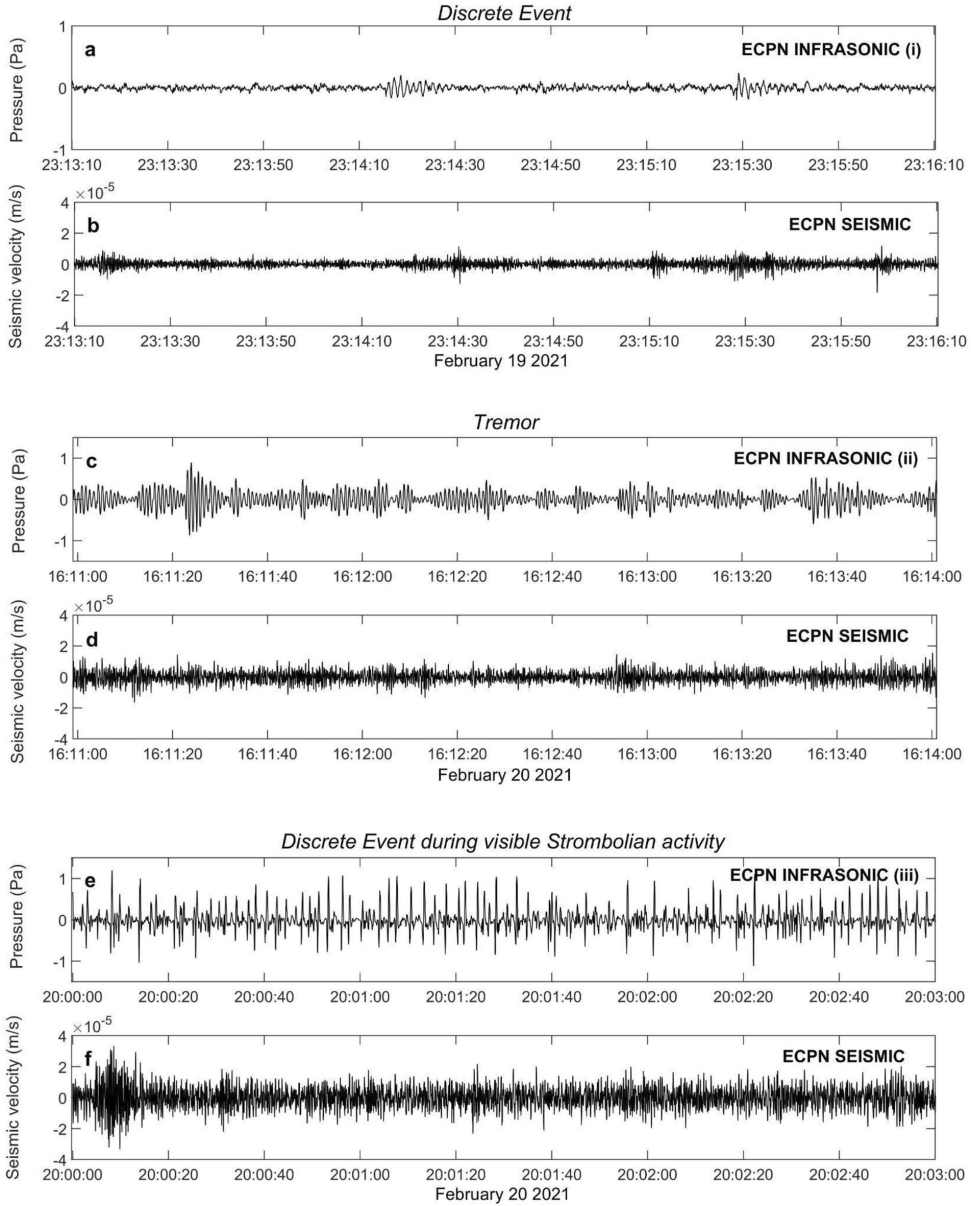
Three minute long infrasound and associated seismic signal examples recorded during: (**a**) and (**b**) the first phase, characterized by discrete infrasound events and no visible subaerial explosion activity; (**c**) and (**d**) the third phase featured by an infrasound event amplitude and occurrence rate increase leading to a continuous tremor; and (**e**) and (**f**) visible Strombolian activity during which discrete explosive events were recorded. The symbols (i), (ii) and (iii) in (a,c,e) are annotated at the appropriate times in Fig. 2a,b.

**Figure 4:**
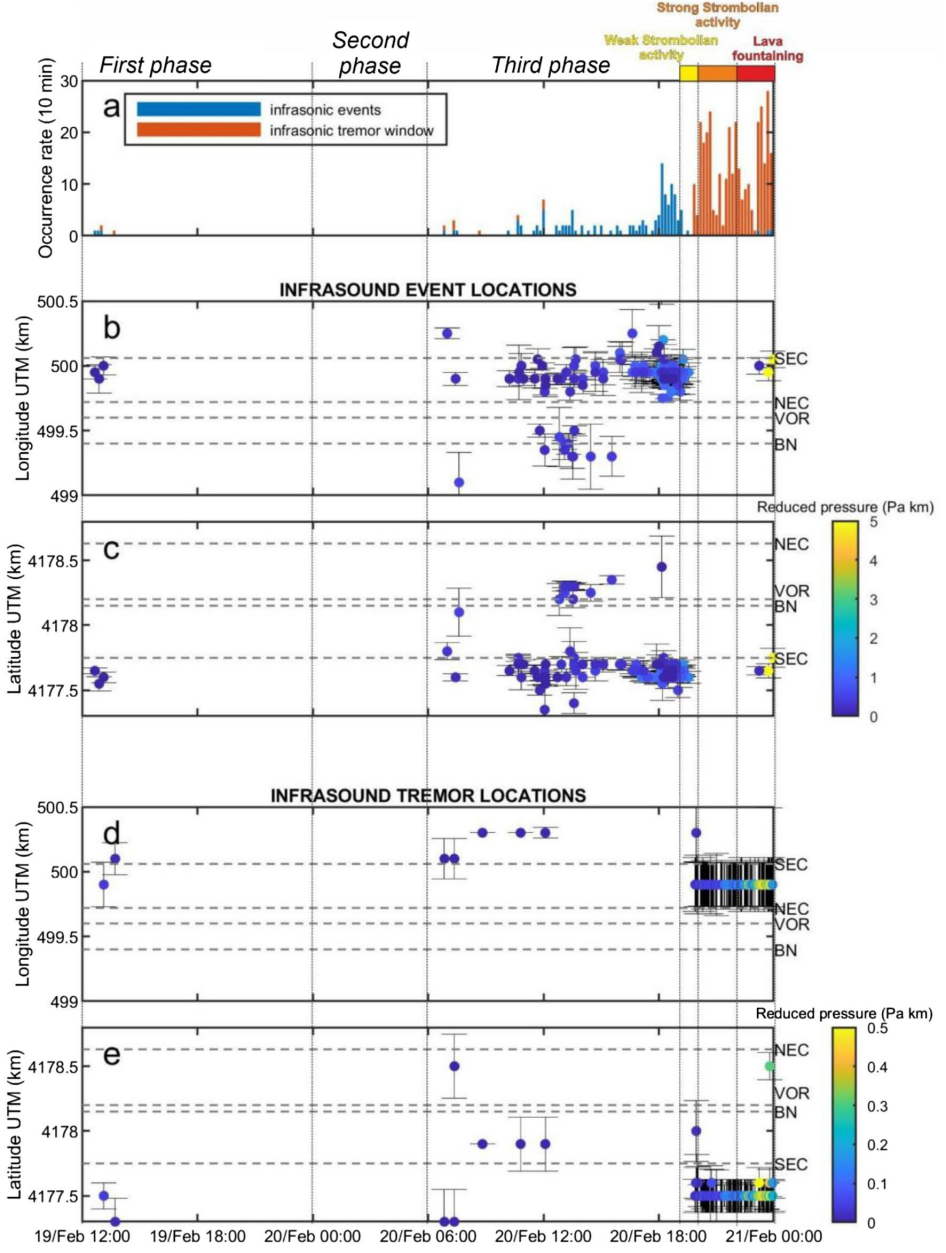
Infrasound source locations. (**a**) Stacked histogram showing the occurrence and timing of both infrasonic events (blue bars) and infrasonic tremor windows (red bars) binned as 10-min time intervals. (**b** and **c**) Longitude and latitude of the located infrasonic events. (**d** and **e**) Infrasonic tremor windows locations. The color of the dots in (b—e) indicates infrasound pressure reduced to a distance of 1 km from the source assuming a spherical spreading model. The horizontal dashed lines show the coordinates for the center of each crater. There are multiple active vents within each crater and vent locations can change with time. The vertical dotted lines show the phases, described in the text, as well as the time intervals characterised by different kinds of visible eruptive activity.

The infrasound data in Figs. 2a,c,e show systematic variation in the 20 h preceding episode #4 with the peak frequency of the monotonic infrasound signal increasing from 0.7 to 3 Hz. Waveform examples of infrasound signal types recorded during this time interval, together with the associated seismic signal, are plotted in Fig. 3. Mount Etna has a complicated summit terrace with several craters sometimes active simultaneously. Because two crater areas (BN and SEC) were active during this period, we perform network infrasound processing, as described in Cannata et al.^23^, to identify the infrasound source location. This analysis confirms that the vast majority of discrete events and tremor were located at SEC (Fig. 4).

Prior to the onset of Strombolian activity, we divide the eruptive activity into three phases, as annotated in Fig. 2.

The first phase is from 12:00 on 19 February (when we start our analysis) to 00:00 on 20 February. Between 12:00 and 14:00 there are several discrete explosive events that were located in the SEC area (Fig. 4). These events are superimposed ontop of a continuous signal, characterized by relatively broadband spectral energy between 0.3 and 1 Hz, and associated with non-volcanic microbaroms generated by ocean noise^24^. The microbarom is distinguishable from the volcanic signal as it is incoherent across the network whereas the low frequency volcanic tremor is coherent. From 21:00 on 19 February to 00:00 on 20 February, a low amplitude monotonic infrasound signal with a peak frequency of 0.7 Hz emerges from the background ocean noise.

The second phase is from 00:00 to 06:00 on 20 February. During this time, the amplitude of the monotonic infrasound signal decreases and the microbarom becomes more evident, particularly on the normalized infrasound spectrogram (Fig. 2e). We exclude this phase from our later analysis in order to avoid misinterpreting the low frequency microbarom as volcanic signal. Note that when observed, the peak frequency of the monotonic infrasound signal is constant at 0.7 Hz during this phase.

**Figure 5:**
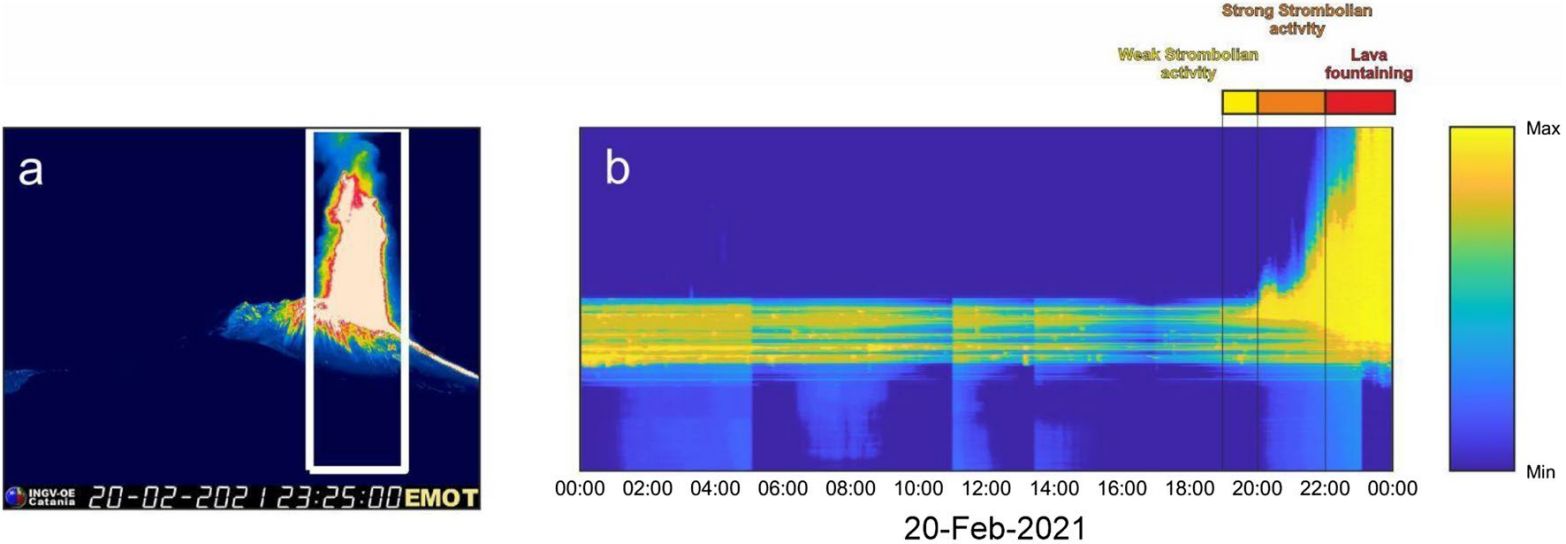
Thermal imagery of lava fountaining. (**a**) Image taken by EMOT thermal camera at 23:25 on 20 February 2021 shows lava fountaining at SEC. (**b**) Time/height diagram shows the time evolution of the maximum temperature as a function of height for the area enclosed by the white rectangle in (**a**). An animation derived from EMOT thermal imagery video and integrated with infrasonic signal is provided in Supplementary Materials (Supplementary Video 1).

The third phases is from 06:00 to 19:00 on 20 February. During this time the infrasound events increased in amplitude and occurred more frequently (Fig. 4) prior to the onset of visible Strombolian activity at 19:00 (Fig. 5). The most significant change, and the focus of this study, is the dramatic increase in peak frequency of the monotonic infrasound signal from 0.7 to 3 Hz.

A comparison of visual and thermal imagery observations with infrasonic signal character helps to illuminate the changing phases of episode #4 (Fig. 5; animation in Supplementary Material). The infrasonic gliding and peaked spectral signal disappeared at about 19:00 on 20 February at the same time as weak Strombolian activity became visible at SEC (INGV, 2021). The explosive activity evolved into strong Strombolian activity an hour later at 20:00 (Fig. 5) and finally into lava fountaining at 22:00^27^. Both transitions were accompanied by a large increase in infrasonic and seismic amplitude (Figs. 2, 3e,f) and the occurrence of a continuous infrasonic tremor (Fig. 4a,d,e).

Joint comparison of seismic data reveals interesting first-order observations (Figs. 2b,d,f and 3). Notably, there is minimal seismic signal with no distinct spectral character prior to the start of Strombolian activity at 19:00. Seismic tremor amplitude increases with the onset of the visual eruption, yet no monotonic signal or spectral gliding is observed. After 20:00, the seismic signal rapidly increases in amplitude and has broad spectral content. The increased spectral amplitude corresponds with the onset of strong Strombolian activity and lava fountaining as observed by thermal imagery (Fig. 5). Joint seismo-acoustic observations, with gliding observed in the infrasound but not in the seismic data (Fig. 3a–d), point to the resonance phenomena occurring in the open crater/conduit of the SEC and coupling well to the atmosphere but not to the ground.

The prominent infrasound signal and lack of seismic signal suggests a source with good coupling to the atmosphere and poor coupling to the solid Earth. Several factors affect the relative partitioning of acoustic and seismic energy such as the location of the explosive/degassing source, the impedance contrasts between magma and the volcano edifice, the source dimension and the conduit conditions^25^. For this eruption, we suspect that the source was subaerial and located very shallow (at the top of the magma column) leading to efficient acoustic radiation but inefficient seismic emission.

This infrasonic gliding observation was common to the signals acquired by all the analyzed infrasonic stations and, as such, is likely indicative of a source process rather than changing propagation conditions.

### Infrasound modeling

We model the gliding monotonic infrasound signal as an acoustic resonance of the crater and conduit, whose length changes with time as magma ascends in the conduit. Acoustic resonance of volcanic craters occurs when acoustic waves, excited by explosions or unsteady degassing at the top of the magma column, are reflected from the crater outlet due to a contrast in the acoustic impedance between the crater outlet and the atmosphere^15,26^. For a narrow cylindrical pipe (ka < < 1 where k is the wavenumber and a is the radius) that is closed at the base and open at the outlet, the resonant frequency, f, is given by:

1 $${\text{f }} = {\text{ c}}/\left( {{\text{4L}}} \right),$$

where c is the speed of sound and L is the length of the resonating cylindrical cavity (i.e., depth from crater outlet to the top of the magma column). Equation (1) predicts an inverse relationship between pipe length and acoustic frequency and provides a rough estimate of expected resonant frequency for a crater with a specific depth. Equation (1) indicates how the infrasound peak frequency rises as magma rises in a conduit and effectively shortens the pipe length. Explosions, which became visible at SEC soon after 19:00 on 20 February^27^, correspond to when the highest infrasound peak frequencies were observed (Figs. 2, 3e,f). This supports the hypothesis that the magma column was high within the crater/conduit at this time.

**Figure 6:**
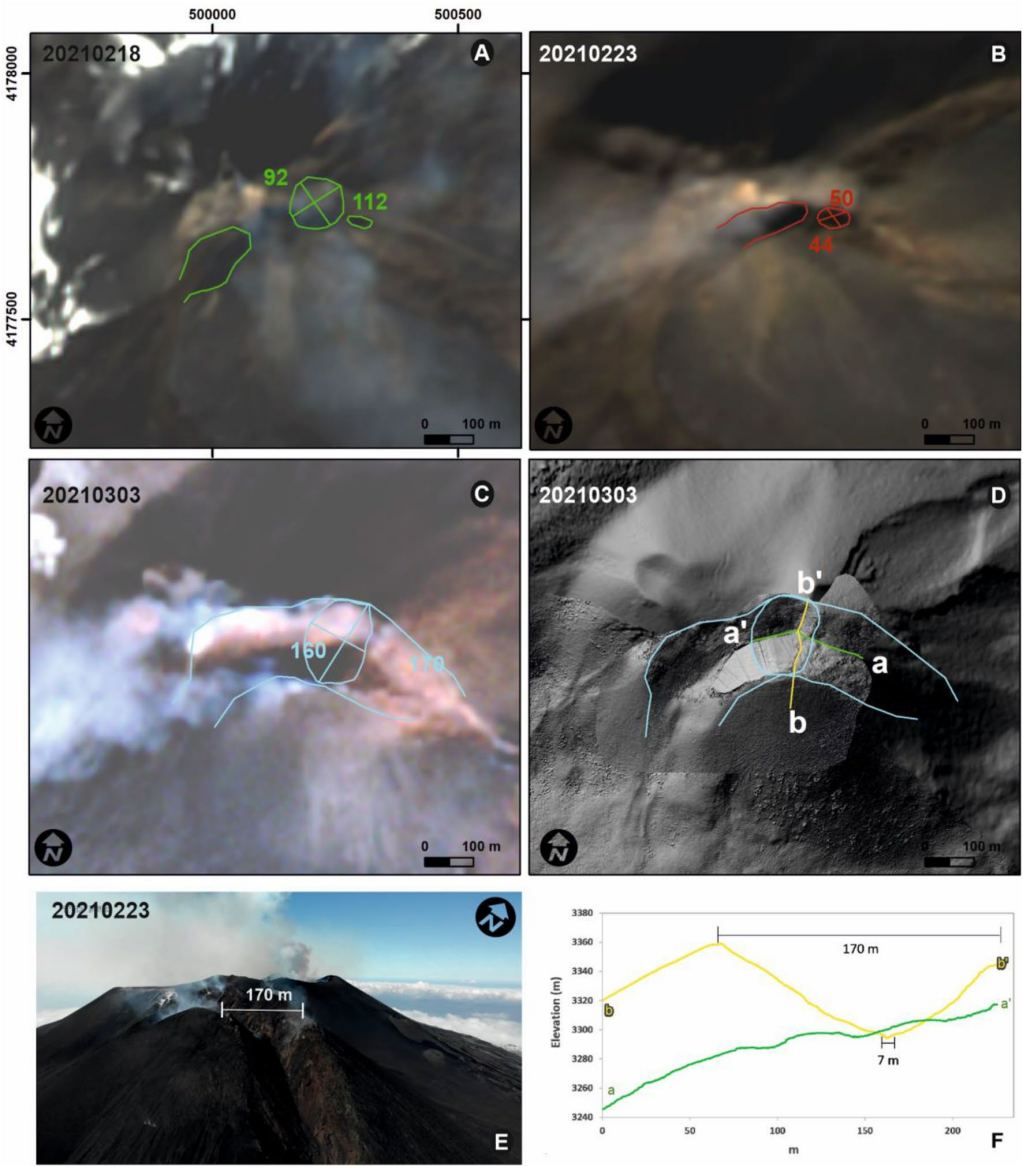
Satellite images (Sentinel-2 L1C) and topography of the SEC. (**a**–**c**) Sentinel-2 L1C images were taken on (**a**) 18 February, (**b**) 23 February and (**c**) 3 March 2021. (**d**) Shaded relief map is obtained from UAS survey on 3 March. The blue colored lines show the crater rim and the breaching on the SEC upper flanks. (**e**) Oblique view image was taken by UAS on 3 March showing the SEC eastern rim and the eastern breach. (**f**) Cross sections show transects in E-W and S–N directions corresponding to green and yellow lines in panel (**d**). Coordinate system UTM WGS 84 33 N. Satellite images were downloaded from https://www.sentinel-hub.com/explore/eobrowser/ with gain, gamma, brightness and contrast adjusted to emphasize the morphological aspects. Afterward, crater rims have been drawn and measured by ArcMap 10.3 https://www.esri.com/en-us/home. Oblique view image shown in (e) was taken by UAS by co-author Massimo Cantarero, the UAS pilot, and the conduit was drawn with CorelDraw X5 https://www.coreldraw.com.

Several previous studies have utilized Eq. (1) to relate harmonic infrasound observations to crater dimensions^21,22,28^, but they have also acknowledged that Eq. (1) is an idealization because volcanic craters are not perfectly cylindrical and/or ka is not far smaller than 1. Hence other studies have used expressions accounting for non-narrow pipes and/or have utilized the analytical solutions for exponential or Bessel horns, to model frequency as a function of crater size^14,29^. More recently, numerical models, both 1D and 3D, have been used to account for more complex geometries^15,20,30,31^. The SEC geometry at Etna is not easily characterized by an analytical expression or geometric approximation. Visual observations of the SEC and structure-from-motion mapping efforts reveal a shallow 65-m-radius crater sloping down towards a vertical conduit with a radius of about 10 m starting about 58 m below the surface. The conduit length is presumed to be variable, changing as magma rises and falls. The coupled crater and conduit, heretofore referred to as the SEC, appears axisymmetric (Fig. 6). The SEC has the appearance of a long-stem funnel and accounts for the system’s resonant frequencies and resonant character.

### Forward modeling

Our analysis uses CRes (Crater Resonance^32^), which is a numerical model for simulating the crater acoustic response for quasi-1D crater geometries, to model the infrasound signal observed at Mt. Etna. CRes solves the linear acoustics wave equation within the crater and models acoustic radiation outside of the crater as radiation from a baffled piston. For more details, refer to Watson et al.^15^. We use the CRes model to calculate the acoustic response, and peak frequency values, of the SEC for varying magma levels. The CRes model has been shown to be a good predictor of crater acoustic response for relatively narrow conduits and craters; a comparison of CRes results with 3D modeling confirming this is provided in Methods.

A digital elevation model of the SEC was reconstructed from topography acquired by an unoccupied aerial system (UAS) survey flown on 3 March 2021 and constrained by satellite observations (Fig. 6), whereas the lava fountaining episode considered here occurred on 20–21 February. The SEC rim has a horseshoe shape, opening toward SE (Fig. 6a), due to a collapse that occurred during the eruptive episode of the 12–13 December 2020. From 21 February, an escarpment was visible in the SE crater rim, likely a consequence of structural weakness due to the numerous fractures that characterized the east flank of the cone. Continued failure and collapse caused the escarpment to deepen and was observed in satellite images from 23 February and 3 March (Fig. 6b,c). We hypothesize that the change in crater geometry meant that the crater could no longer efficiently support acoustic resonance and this is one of the reasons why only the fourth lava fountain exhibited gliding (see section Discussion).

Allard et al.^33^ has reported magmatic temperatures of ~ 1100 °C at Mount Etna, however entrainment of ambient air means that the temperature within SEC will likely only be several hundreds of degrees celsius. This is supported by the temperature measurements performed by Fee et al.^12^, who estimated temperatures of 200 °C within the gas-filled cavity of the Halema’uma’u Crater (Kilauea Volcano, Hawaii), and by Sawyer et al.^34^ , who retrieved volcanic gas temperatures in the range 27°–267 °C above the lava lake in Nyiragongo (Congo). In previous work on monotonic infrasound signals, Johnson et al.^28^ combined analytical expressions for the peak frequency and quality factor of a resonating pipe to estimate a constant air temperature within the crater at Cotopaxi (Ecuador). However, it is unclear how this approach can be applied to complex crater geometries or a spatially variable temperature profile.

We do not have any direct observational constraints on the air temperature within the crater/conduit or its variation with depth. The air temperature within the crater/conduit will increase as magma ascends in the crater, however, we note that Watson et al.^15^ demonstrated that a spatially varying temperature profile has a minimal impact on the peak frequency (Supplementary Fig. 3). Hence, rather than trying to account for the second-order effects of spatially variable and changing air temperature, we assume a constant air temperature of 200 °C. The speed of sound is calculated by

2 $$c = \sqrt{\gamma RT}$$

where c is the sound speed, $\gamma$ is the ratio of heat capacities ($\gamma$=1.4 for an adiabatic process), R is the specific gas constant, and T is the temperature in Kelvin.

Based on INGV observations (personal communications), we assume an atmospheric temperature of −7.5 °C. We examine the sensitivity of the results to the assumed temperature profile in the Supplementary Material.

**Figure 7:**
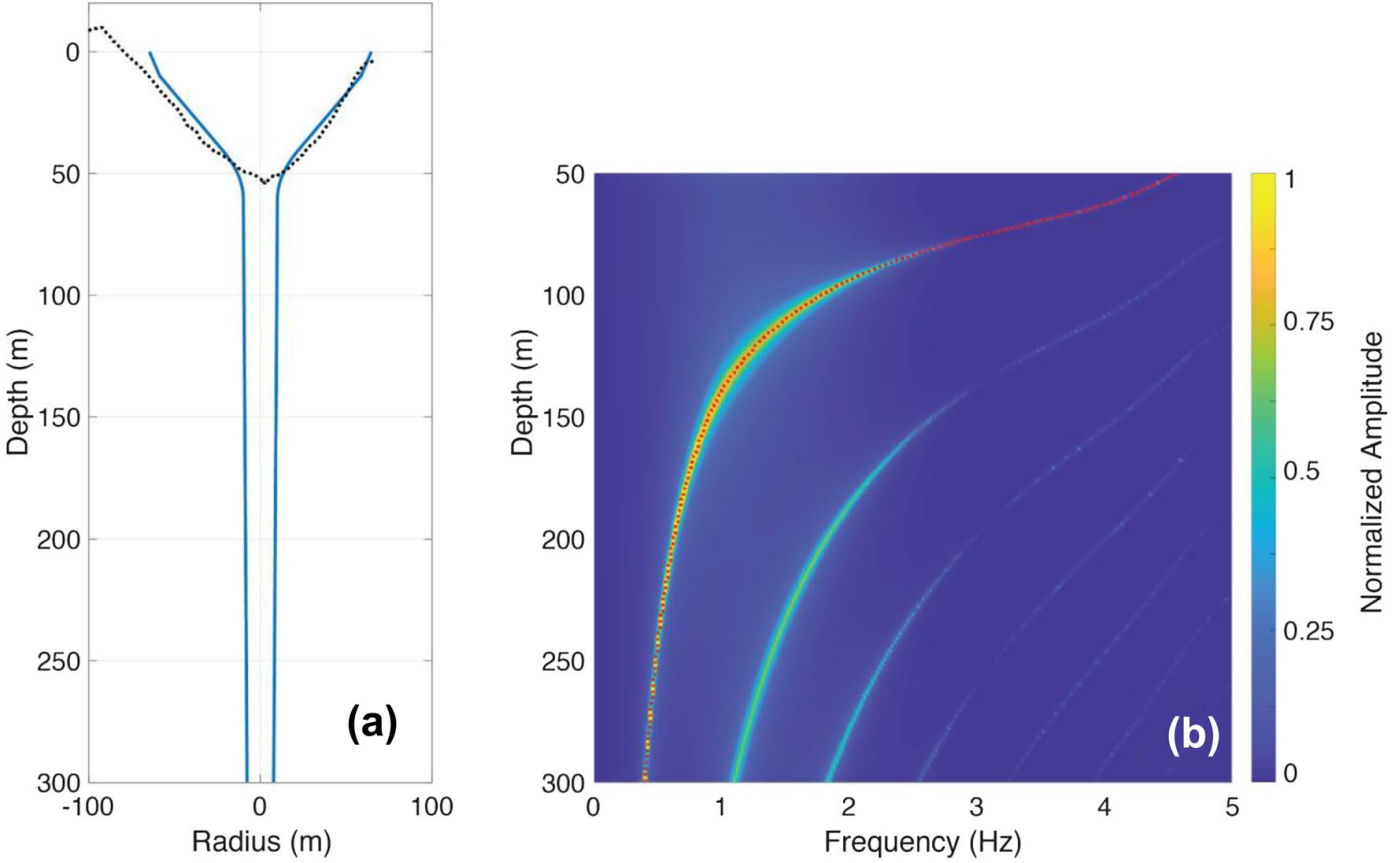
Forward simulations of CRes showing spectra of SEC acoustic response function for varying magma levels. (**a**) The model geometry indicates the cross-section through the digital elevation model of the crater (black, dotted) and the fitted 1D axisymmetric geometry used in modeling (blue, solid). (**b**) Spectral responses, as a function of depth, are shown with normalized amplitudes of infrasound signal. Red line indicates the modeled peak frequency.

We use CRes to simulate the acoustic response function for sources located at the bottom of SEC where SEC depths range from 300 to 50 m at 1 m intervals, reflecting possible magma depths in the conduit. Figure 7b shows the normalized spectrogram of the SEC response as a function of magma level. As expected, it shows systematic variations in both the fundamental mode and the overtones. The acoustic response function is the impulse response of the crater geometry^15^. In contrast, the recorded infrasound signal (Fig. 2c,e) is the time-domain convolution of the acoustic response function with a source function. Compared to an impulse source that has equal energy at all frequencies, realistic source functions have limited energy at high frequencies^15^. CRes only accounts for damping through acoustic radiation whereas there are additional sources of damping in nature, such as intrinsic attenuation and coupling of acoustic waves to the solid Earth. Furthermore, higher frequency signals are more rapidly attenuated with distance. This explains why only the fundamental peak is visible in the data whereas there are multiple overtones seen in Fig. 7b. While the choice of source function strongly influences the overtones, the fundamental model is relatively unaffected^15^ (Supplementary Figure 2). Therefore, rather than assuming an arbitrary source function, we perform our analysis with the acoustic response function.

The results from CRes 1D modeling confirm a monotonic relationship between magma level and frequency (Fig. 7b), which allows for frequency to be used as a unique proxy for SEC depth. Because the frequency changes are significant and correspond to magma surface within a deep conduit the analysis does not require incorporation of an infrasound quality factor used in other studies^19,28^.

### Inversion

**Figure 8:**
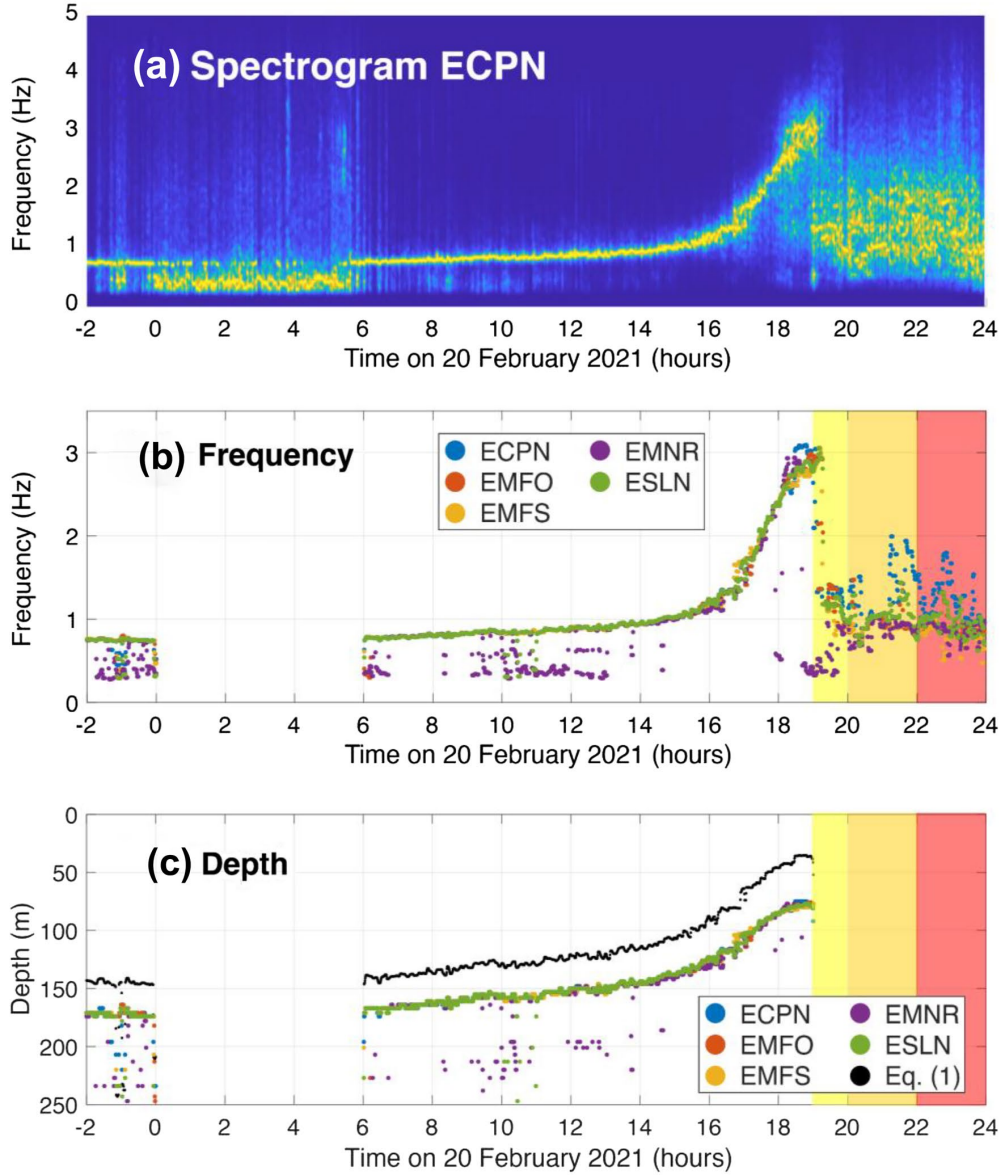
(**a**) Normalized spectrogram from ECPN showing the monotonic infrasound signal and spectral gliding. (**b**) Peak frequency for the five infrasound stations with the best signal-to-noise ratio; (blue) ECPN, (red) EMFO, (yellow) EMFS, (purple) EMNR, and (green) ESLN. (**c**) Inverted depth from the crater outlet to the top of the magma column. Black circles show the inverted depth for Eq. (1) with c = 436 m/s. Colored boxes indicate different phases of the eruption: (yellow) weak Strombolian activity, (orange) strong Strombolian activity, (red) lava fountaining.

The CRes simulations produce an unique relationship between peak frequency and depth (Fig. 7b). Based on this information, we can relate the observed peak frequency at each time (Fig. 8b) with a depth value (Fig. 8c) in order to invert the infrasound observations for the depth. During phase 2 (00:00 to 06:00 on 20 February) the volcanic signal decreased in amplitude and the microbarom dominated the infrasound recordings, hence we focus our analysis of phases 1 and 3 when there are clear volcanic signals recorded.

**Figure 9:**
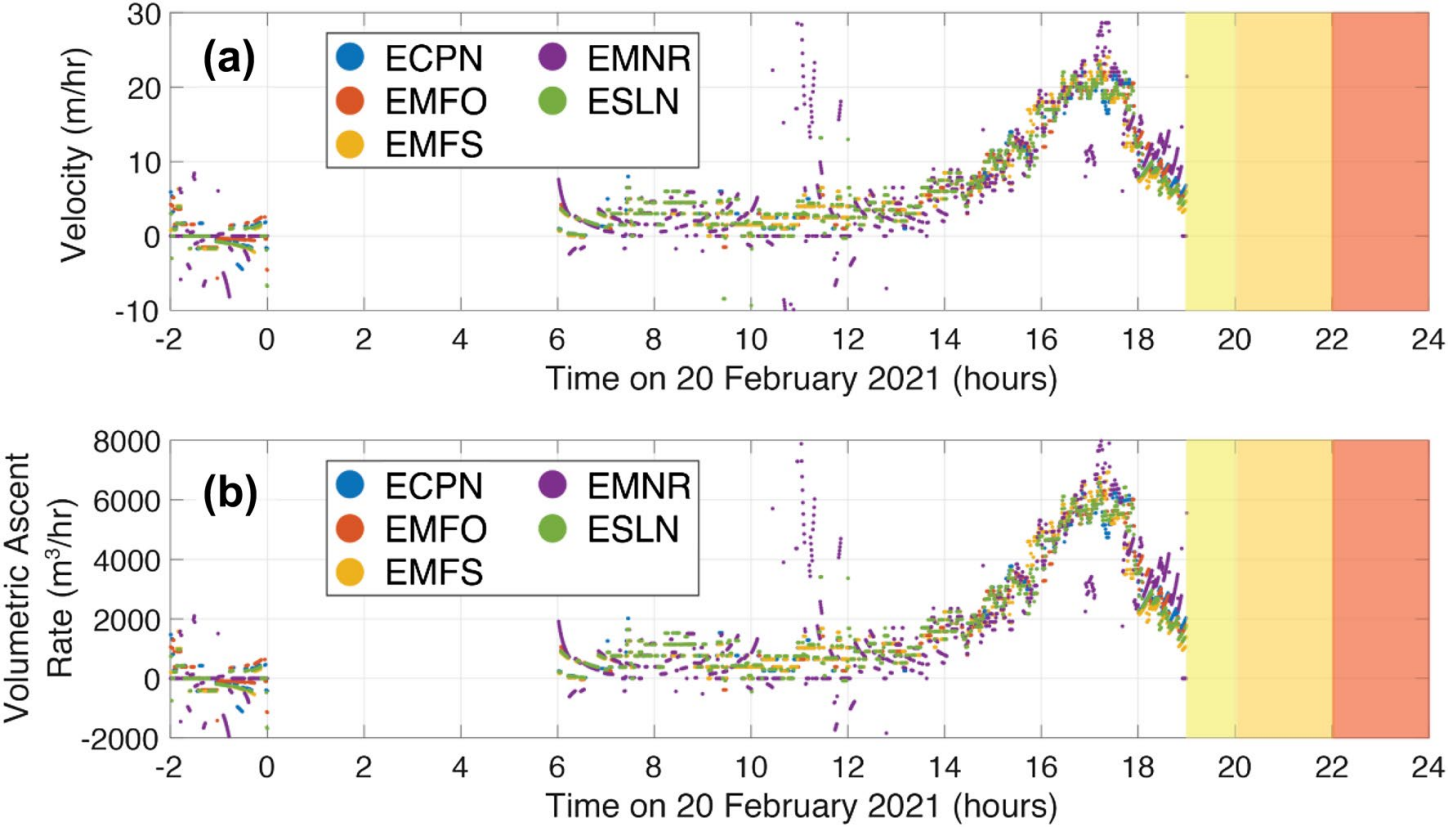
(**a**) Magma ascent rate (m/hr) and (**b**) volumetric ascent rate (m^3^/hr) for the five infrasound stations with the best signal-to-noise ratio; (blue) ECPN, (red) EMFO, (yellow) EMFS, (purple) EMNR, and (green) ESLN. Colored boxes indicate different phases of the eruption: (yellow) weak Strombolian activity, (orange) strong Strombolian activity, (red) lava fountaining.

The spectral evolution of the recorded infrasound (Figs. 2, 8a,b), indicates a systematic increase in frequency that can be attributed to rising magma column and decreasing depth of SEC (defined as the distance from crater rim to inferred magma level). Starting at 22:00 on 19 February, the magma column appears to be deep (170 ± 25 m) within the conduit. From 00:00 to 06:00 on 20 February, infrasonic volcanic activity decreased to the point where the microbarom peak dominates and the 0.7 Hz tremor is only intermittently evident (Fig. 8a). From 06:00 to 19:00 the peak frequency increases, slowly at first and then more rapidly after 15:00. This is modeled as a depth decrease from 167 ± 25 m to 138 ± 19 m over nine hours (average velocity of 3.2 m per hour) and a decrease from 138 ± 19 m to 78 ± 8 m over the course of four hours (average velocity of 15 m per hour) (Figs. 8c, 9a).

In addition to the CRes inversion, we invert the peak frequency for the depth using Eq. (1) with c = 436 m/s, which corresponds to a temperature of 200^O^C (Fig. 8c). Equation (1) describes the geometry as a cylindrical pipe and does not account for the flaring crater geometry (Fig. 7a). Despite this simplified geometry, the inverted depths using Eq. (1) are qualitatively similar to the CRes results (~ 30 m shallower) and show the same pattern of accelerating magma ascent prior to the onset of lava fountaining. While the CRes inversion is computationally efficient, it can take significant time to determine the various inputs parameters, particularly the crater geometry. In contrast, Eq. (1) is a simple model that only requires sound speed as an input parameter but produces qualitatively similar results. Equation (1) can easily be calculated in real-time (either for a single sound speed or for a realistic range of values) and would be straightforward to incorporate this analysis into existing monitoring workflows. We note that there is reasonable agreement between Eq. (1) and the CRes inversion for the crater geometry considered here but this may not be the case at other volcanoes with different geometries.

The monotonic infrasound signal disappears after 19:00 as the eruption transitions to Strombolian activity (Fig. 3e,f) and lava fountaining with broadband infrasound signal (Fig. 8a). Therefore, after 19:00 we can no longer invert the peak frequency for the depth. We attribute the loss of resonance to the magma level reaching the flaring crater of SEC where open conduit resonance is no longer sustained.

We use a moving median with a sliding window of 2 h to smooth the inverted depths and then differentiate to calculate the magma ascent velocity as a function of time (Fig. 9a). The volumetric ascent rate is estimated by multiplying the ascent velocity with the cross-sectional area of the crater (Fig. 9b). The maximum volumetric ascent rate is ~ 6300 m^3^/hr and the mean value is ~ 1800 m^3^/hr, which is approximately 0.5 cubic meters per second. The calculated volumetric ascent rates are sensitive to the assumed geometry and cross-sectional area of SEC. While uncertainty of the conduit radius is difficult to quantify, we note that volumetric ascent rate scales with the square of the radius meaning that small errors in the conduit radius lead to large errors in the volumetric ascent rate. Nonetheless, the numbers presented here provide a useful illustration of how infrasound can constrain both magma rise rates and volume fluxes.

## Discussion

### Acoustic resonance

The monotonic infrasound signal, recorded before the onset of the 20 February lava fountain (Fig. 2), suggests strong acoustic resonance of the volcanic crater, which has been previously observed at Mt. Etna^13,20–22^. The lack of seismic signal accompanying the featured monotonic infrasound signal indicates that the source occurs in a conduit-like volume with good coupling to the atmosphere and relatively poor coupling to the solid Earth. We hypothesize that acoustic waves are generated by explosions and unsteady degassing at the magma free surface.

The resonant frequency of the radiated infrasound is similar at all stations (Fig. 8b) indicating that the observations are due to lumped source and SEC response effects, as opposed to propagation effects outside the SEC or site effects local to each station. Previous work at other volcanoes has demonstrated the possible influence of topographic scattering for local infrasound observations^35–37^, however the remarkable consistency in peak frequency observations here indicates that topographic effects are a second-order influence (at least for the low frequencies (< 3 Hz) considered here).

Infrasonic gliding is a particularly remarkable feature of the monotonic signal featured in this study. Although gliding has been reported for volcano seismic tremor, the observations of gliding in the volcano infrasound wavefield are limited. Spectral shifts in the infrasound wavefield have been mentioned in a few studies, such as at Arenal^38^, Pu`u `Ō`ō^39^ and Tungurahua^40^, however they entailed only a brief duration, occurred before or after single explosions and/or were characterized by both the increase and the decrease of peak frequencies^38,40,41^. Shifting in the spectral peaks of the harmonic tremor has usually been attributed to variation in speed of sound in the fluid (assuming acoustic resonance)^38^ or it has been related to repeated low-frequency events or ‘chugging’ phenomena, where harmonic infrasound is accompanied by audible pulsing^41^.

Etna’s long-duration (~ 20 h) evolution in monotonic tremor and systematic increase in frequency is perhaps most analogous to infrasonic frequency changes observed at Villarrica (Chile) in the 5 days leading up to its 3 March 2015 lava fountaining episode. At Villarrica the fundamental frequency of discrete explosion signals rose from 0.7 to 0.95 Hz (30% increase) and was accompanied by a drop in coda resonance as the lava free surface rose, first within a pipe-like conduit and then up into the broader crater^19^. As with the episode at Etna, the shallow level of the magma free surface preceded observable Strombolian activity and then, a few hours later, a vigorous lava fountain 1.5 km high.

The frequency change at Etna between 19 and 20 February was far more rapid and dramatic (from 0.7 to 3 Hz; 400% change) than at Villarrica, occurring over the course of just 18 h. The higher resonant frequency supported by Etna’s SEC, compared to Villarrica, is explained by resonance of a narrower pipe (10 m compared to 30 m radius) and smaller size of crater (less than 60 m deep at Etna). Etna’s infrasound reached a maximum value of 3 Hz at about 18:00 on February 20th and stabilized at this level for around an hour, before disappearing. Our depth inversion suggests that the magma is high and has possibly reached the flaring portion of the crater at this time (Fig. 8c). As the magma rises and the vertical conduit disappears so does the resonance. Strombolian activity after 19:00 and ensuing lava fountaining are characterized by broadband infrasound signals (Fig. 8). Our interpretations are supported by the results obtained in Spina et al.^42^, who performed analogue degassing experiments and simulated harmonic acoustic tremor. They recorded audio frequencies in a resonating conduit as they modified the level of the silicone oil (analogue magma) through which gas bubbles were permitted to rise and burst.

**Figure 10:**
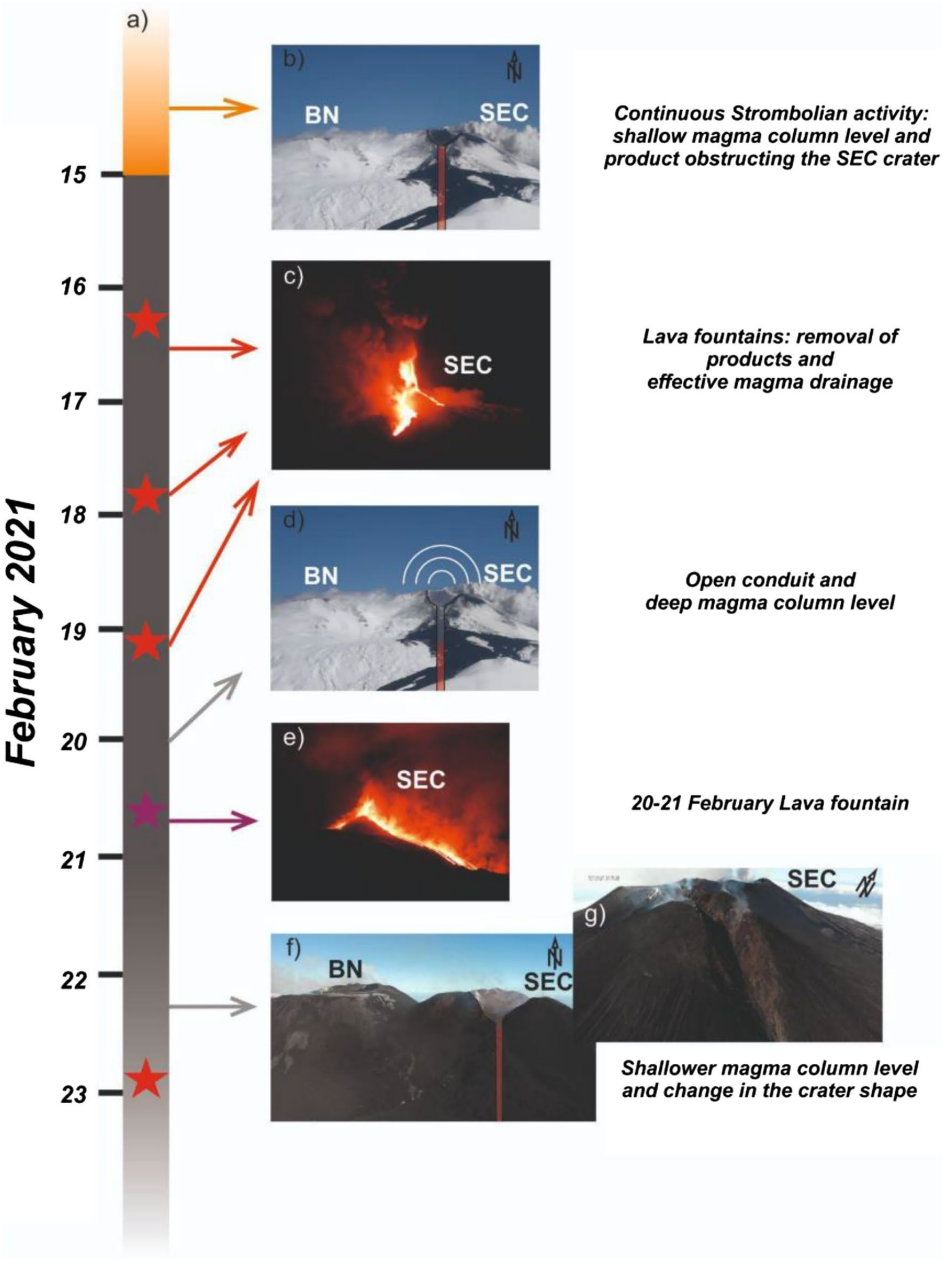
(**a**) Timeline of the volcanic phenomena preceding and following the observed gliding signals, with red stars indicating lava fountain episodes in the time period 16—19 February, and purple star the 20 February lava fountain episode (episode #4), which is the focus of this study; (**b**) photo of Etna summit crater with a schematic cartoon of the shallower portion of SEC feeding system representing hypothesized conduit condition and magma level before the onset of the paroxysmal activity period; (**c**) photo of the 16 February lava fountain (courtesy of Boris Behncke); (**d**) photo of Etna summit crater with a schematic cartoon of the shallower portion of SEC feeding system representing hypothesized conduit condition and magma level leading to gliding phenomenon; (**e**) photo of episode #4 (courtesy of Boris Behncke); (**f**) photo taken on 3 March of Etna summit craters with a schematic cartoon of the shallower portion of SEC feeding system representing hypothesized conduit condition and magma level after episode #4 and g) photo showing SEC shape after 22 February. Images b,d,f and g were taken by co-author Massimo Cantarero, the UAS pilot.

A principal question is why only episode #4 was preceded by a very evident gliding phenomenon (Fig. 10). To support acoustic resonance a particular geometry is needed as well as a source phenomenon that excites acoustic waves^15^. In the months before the first lava fountain, SEC was affected by almost continuous Strombolian activity^43^ (Fig. 10a,b). We speculate that the three previous lava fountain episodes (16, 17–18 and 19 February) had no obvious monotonic gliding signal because the magma column level was shallow during these episodes, which prevented effective resonance (Fig. 10a,b). The first three lava fountains emitted a total volume of 10 Mm^3^^27^, which is much larger than the average volume of 1.1 Mm^3^ for typical lava fountaining episodes at Mt. Etna^44^. We speculate that these large discharges drained magma from the conduit so that prior to episode #4 the magma level was deep in the conduit, which allowed for efficient resonance and the observation of a very evident infrasonic gliding as the magma column rose prior to lava fountaining.

Concerning the absence of gliding in lava fountains following episode #4, after the 22 February episode, the summit area of the SEC changed significantly and a horseshoe-shaped scar was formed by the coalescence of multiple active vents during the lava fountains and partial collapse of the crater wall (Fig. 10a,f,g). The destructive nature of the waning portion of episode #4 is evident in the thermal video (Supplementary Video 1). We propose that changing conduit and crater properties inhibited the development of resonance during successive lava fountain episodes. Furthermore, we speculate that during the other episodes where gliding was absent, the magma level might not have been deep enough with SEC and/or the resonance excitation mechanism (bubble bursting and degassing) might not have been energetic enough to produce resonance. We note that the focus of this research is investigating the gliding phenomenon and constraining magma movements, rather than exploring the precise source mechanism that causes the acoustic resonance, which we hope will be the goal of future studies.

### Magma ascent

The ascent position over time can be used to calculate the magma ascent rate and the associated volumetric flux into the conduit (Fig. 9). This result represents one of the first examples of using infrasound to constrain magma rise rates leading to an eruption. In previous work, Richardson et al.^14^ used infrasound and seismic data to examine changes in the lava lake level at Villarrica over a period of weeks. Sciotto et al.^13^ and Spina et al.^21^ related changes in infrasound peak frequency at Mt. Etna to changes in magma column height and pressure in the magma chamber in time periods preceding and/or following eruptions. Watson et al.^20^ previously examined harmonic infrasound observations at Mt. Etna during the “Christmas Eve” 2018 eruption and inferred a decrease in the magma column accompanying a flank eruption. Watson et al.^20^ focused on two time windows (before and after the flank eruption) and hence does not have the same temporal resolution as we obtained here. Johnson et al.^19^ tracked the ascent of magma prior to a paroxysmal eruption at Villarrica using infrasound data from a single station. While Johnson et al.^19^ calculated the depth to the magma column, they did not explicitly calculate the ascent rate or the volumetric flux. Furthermore, the change in peak frequency at Villarrica was an order of magnitude less than observed in this study.

Infrasound-derived depths of the magma surface hint at a mean ascent rate of 5 m/hr and a maximum rate of 22 m/hr. This is an order of magnitude greater than the rate observed by Johnson et al.^19^ at Villarrica where the magma column rose 70 m in 3 days (February 26th to March 1st), which gives an average ascent rate of ~ 1 m/hr, or 3000 m^3^/hr (~ 1 m^3^/s assuming a 30 m conduit radius). The narrower conduit of Etna’s SEC (~ 10 m; area of ~ 300 m^2^) leads to mean ascent rates of ~ 1450 m^3^/hour to a peak value of 6200 m^3^/hour (~ 2 m^3^/s). Notably, these volumetric rates may be affected by the variable density of the magma, which could be high (volatile-poor) or relatively low if the magma is gas-rich. The volumetric rates are also highly sensitive to the assumed conduit radius. The apparent acceleration of the rising magma surface could potentially be explained by a constant mass flux and increasing frothiness directly prior to the Strombolian activity.

Our ascent rates are mainly lower than the range of ascent rates (10^–2^–10^2^ m/s) of basaltic melts inferred from methods such as isotopic-decay dating, crystal-size distribution analysis, melt-inclusion timekeepers, chemical-diffusion-based geo-speedometers, thermal modeling of magmatic systems, mechanical modeling of magma ascent and seismicity studies (Petrelli et al.^45^ and references therein). Our observations relate to the bulk movement of magma whereas other methods, such as melt-inclusion timekeepers and geo-speedometers, relate to the movement of individual crystals which may ascend faster than the bulk magma, potentially explaining why our observations are lower than other estimates of ascent rates. Furthermore, we note that we are analyzing the ascent of magma in the upper portion of an open conduit whereas many of these methods constrain ascent rates of dykes or other magma movement in the crust. Care should be taken when comparing ascent rates calculated for different phenomena.

Mt. Etna is one of the most well-monitored volcanoes in the world. In addition to the seismic, infrasound, and thermal camera data presented here, Mt. Etna is monitored with geodetic instrumentation consisting of tiltmeters and GNSS stations. The GNSS data can provide useful information about deformation and magma recharge during a sequence of lava fountains^46,47^. The uncertainties in the GNSS measurements, however, are generally too high to reliably measure deformation during a single paroxsym. Tilt data have higher temporal resolution and show that clear changes take place during each paroxsym from the summit vents^47,48^. While the deflation pattern due to eruption of magma from the summit is often clearly observed, the inflation pattern preceding eruptions is frequently weaker. For the paroxsym that we focus on here, the inflation pattern was only observed at a single tiltmeter, which means that the location and properties of the inflation source cannot be reliably constrained.

### Perspectives and outlook

Open vent volcanoes like Mt. Etna are some of the most frequently erupting and hazardous volcanoes on the planet. Here we highlight a paroxysmal lava fountaining episode that was preceded by infrasonic gliding, where the peak frequency of monotonic infrasound signals increased in the days and hours prior to the eruption. We demonstrate how infrasound analysis, acoustic resonance modeling and high-resolution topographic surveys can be combined to track magma movements within the shallow plumbing system during infrasonic gliding. In particular, we place quantitative constraints on the magma depth, its ascent velocity and volumetric flux. The approach shown in this paper allows the magma dynamics in open vent volcanoes to be reconstructed in high temporal resolution.

During a volcanic crisis, time constraints mean that it is unlikely that observatory staff will be able to replicate the sophisticated topographic analysis and infrasound modeling shown here. Therefore, we demonstrate that the simple analytical model of an open-closed pipe can be used to produce qualitatively similar results (for the geometry considered here). We suggest that this technique could be applied at open vent volcanoes around the world to analyze infrasonic gliding and obtain qualitative constraints on magma movement in real-time.

The observations of infrasonic gliding prior to lava fountaining that are presented here and in previous work at Villarrica^19^ suggest that infrasonic gliding could be a precursory signal to lava fountaining under certain conditions. However, infrasonic gliding was only observed prior to one of the 52 lava fountaining episodes that occurred during the eruptive sequence of 2021 at Mt. Etna. In order for infrasonic gliding to be used as a forecasting tool, future work is needed to (1) better understand the conditions under which infrasonic gliding occurs, (2) determine the probability of lava fountaining occurring after infrasonic gliding (compared with the probability of infrasonic gliding occurring with no subsequent lava fountaining), and (3) develop a forecasting model that uses the infrasound-derived depth information as an input and outputs the likelihood of lava fountaining occurring. While we focus on lava fountaining in this study, infrasonic gliding could plausibly precede other styles of eruptive activity (i.e., effusive lava flows). Further observations of precursory infrasonic gliding are needed to determine if characteristics of the infrasonic gliding can be used to estimate the style of subsequent eruptive activity.

## Methods

### Volcanological context

On 24 December 2018 the violent “Christmas Eve eruption” began at Mt. Etna. The eruption lasted for three days during which a 2-km-long fissure opened on the south-east flank of the volcano^22,49,50^. From the end of this eruption until the end of 2020, Etna experienced a period of intermittent and energetically variable Strombolian activity, ash emission and lava flows. This activity took place from all of the summit craters (Fig. 1). Energetic and continuous activity was observed at Voragine (VOR), where, from September 2019 to May 2020, medium-to-high intensity Strombolian explosions and small lava flows took place. The SEC area was intermittently active in May–July 2019, while from December 2019 Strombolian explosions and weak ash emission were continuously observed at summit vents and on fractures opened on its flanks^51^. In April 2020 volcanic phenomena intensified, episodes of sustained Strombolian activity (late May) and a small lava fountain (19 April) occurred. From December 2020 and until the beginning of the lava fountain sequence on 16 February 2021, activity was focused at SEC with a few lava fountains, sustained Strombolian activity, small pyroclastic flows and lava effusion, the most significant of which occurred on 13, 21 and 22 December 2020 and 18 January 2021.

### Crater geometry reconstruction

A UAS (unoccupied aerial system) topographic survey was performed on 3 March 2021. The UAS took off from the top of the 2002–2003 eruption scoria cone (1950 m a.s.l.; Fig. 1b). Sixty images were taken from an average altitude of 400 m, and 8 videos were recorded around the SEC, among these the most meaningful is shown as supplementary material (Supplementary Video 2). The survey covered an area of ~ 1 km^2^ and resulted in a digital elevation model (DEM) pixel resolution of 0.55 m and an orthophoto resolution of 12.9 cm. Structure-from-motion reconstructions using Agisoft Metashape produced a dense cloud of 2,821,217 points and a DEM with 0.55 m resolution and georegistration to the WGS 84 / UTM zone 33 N coordinate system.

Georeferencing by the UAS GPS in areas with steep slopes can have large uncertainties (> 50 m). Photogrammetry best practices suggest spreading ground control points evenly throughout the survey area, which is impossible to do on an active volcano. In order to get accurate georeferencing, we aligned the 2021 DEM with the 2014 DEM^52^ using the unchanged southern flank of the BN summit cone. This correction results in inaccurate absolute elevation values but allows for the calculation of relative distances, such as crater rim size and shape, which are the important values for our infrasound modeling.

The DEM produced from the UAS survey were compared with satellite images from Sentinel 2LC with a resolution ranging from 6.5 m to 8.9 m acquired on 18 and 23 February 2021 and 3 March 2021 (the same day as the UAS survey; Fig. 6). The satellite data were used to constrain the dimensions of the outer crater rim and confirm the results of the UAS survey. However, due to their lower resolution, the satellite data were not used to constrain the geometry of the inner crater. Instead, the 3 March 2021 UAS survey was compared with several other surveys performed over the SEC area after similar eruptive activity.

The UAS survey was collected on 3 March 2021 whereas lava fountain episode #4 occurred on 22 February 2021. The summit area of the SEC changed significantly after episode #4 and a horseshoe-shaped scar was formed (Figs. 6 and 10). We compare the UAS survey with satellite images from 18 and 23 February (Fig. 6a,b) to estimate the crater geometry during the period of interest. As a result, we use the north–south transect (b-b’) shown in Fig. 6d to construct the quasi-1D crater geometry used in our modeling.

### Geophysical data

Seismo-acoustic data was recorded by the permanent monitoring network run by Istituto Nazionale di Geofisica e Vulcanologia – Osservatorio Etneo (INGV-OE; Fig. 1). Infrasound data was recorded at 10 stations, equipped with a GRAS 40AN sensor (microphone) with a sensitivity of 50 mV/Pa. Infrasound data have a flat response between 0.3 Hz (instrument roll-off) and 20 kHz (+ /−2 dB) and recorded at 50 Hz with a 25 Hz Nyquist frequency. Seismic data was recorded by co-located broadband (40 s), three-component Trillium Nanometrics seismometers, acquiring at a sampling rate of 100 Hz with a flat response between 0.025 and 50 Hz. We analyzed infrasound data recorded by stations ECPN, EMFO, EMFS, EMNR and ESLN as they had the best signal-to-noise ratios. These stations range in distance from 1.3 to 8.1 km from the active vent.

Seismic and infrasound spectra are calculated within 81.92 s long windows (2^12^ or 2^13^ samples for infrasonic and seismic signals, respectively) and averaged over 10 min time windows (Fig. 2). Infrasound events and infrasonic tremor windows are first detected from the continuous signal by means of STA/LTA and cross-correlation based trigger algorithm, respectively^23,53^. Once extracted, the source locations for the infrasound signals were computed by grid-search methods based on the computation of brightness function for the infrasonic events and semblance function for the infrasonic tremor^23,53^ (Fig. 4), while event amplitude is expressed by pressure values reduced to 1 km from the source. The source location analysis was performed by using signals recorded by the whole infrasonic permanent network composed of 9 stations (Fig. 1).

Uncertainty in the locations was estimated by the jackknife technique^54^, which is also called “leave one out”. The location algorithm is repeated *n* times where *n* is the number of stations (there are 9 infrasound stations in the permanent network; Fig. 1) with a different station left out of the analysis each time. The error values are calculated by using the *n* location results, as well as the location results constrained by the whole network. More details can be found in Sect. 3.1 of Cannata et al.^23^. In Fig. 3 we only show the relatively well constrained events with location uncertainties of less than 250 m.

### Thermal imagery

We have incorporated thermal imagery analysis from the thermal camera located at station EMOT (yellow square in Fig. 1). To track the evolution of the explosive activity over time, we followed the method proposed by Gaudin et al.^55^ and processed the thermal images. For frame regions cropped above SEC we calculated the maximum temperature as a function of height. The time/height diagram (Fig. 5) then shows the normalized temperatures and provides a qualitative record of when Strombolian activity and lava fountaining commence.

### Uncertainty analysis

Depth inversion from infrasound frequency content is subject to several possible sources of uncertainty. We consider the effects of four primary assumptions including model choice, uncertainty in topographic terrain, source spectrum, and conduit temperature. The uncertainty analysis is described in the Supplementary Materials.

## Supplementary Information

Supplementary Video 1.

Supplementary Information 1.

Supplementary Video 2.

## Data Availability

In accordance with INGV's data policy, infrasound data are publicly available at https://doi.org/10.13127/etna_infra/raw_20210219_20 and seismic data (belonging to the Italian Seismic Network, code IV) can be downloaded through EIDA database (European Integrated Data Archive http://eida.rm.ingv.it/). The latest version of the modeling code CRes is hosted at https://github.com/leighton-watson/CRes and the version used in this paper is archived at https://doi.org/10.5281/zenodo.3235682.
