# Supplementary material for: Infrasonic gliding reflects a rising magma column at Mount Etna (Italy)
Source: Sci Rep. 2022 Oct 19;12:16954. doi: 10.1038/s41598-022-20258-9 (PMC9582027; doi:10.1038/s41598-022-20258-9)
Supplement: Supplementary file 2 — Supplementary Information 1. [file 41598_2022_20258_MOESM2_ESM.pdf]

Supplementary Material for “Infrasonic gliding reflects a rising magma column at Mount Etna (Italy)’

Authors: Mariangela Sciotto[1], Leighton M. Watson[2], Andrea Cannata[1,3], Massimo Cantarero[1], Emanuela De Beni[1], Jeffrey B. Johnson[4]

1. Istituto Nazionale di Geofisica e Vulcanologia, Osservatorio Etneo, Catania, Italy,
2. School of Earth and Environment, University of Canterbury, Christchurch, New Zealand
3. Dipartimento di Scienze Biologiche, Geologiche e Ambientali - Sezione di Scienze della Terra, Università degli Studi di Catania, Catania, Italy
4. Department of Geosciences, Boise State University, Boise, ID, USA

Depth inversion from infrasound frequency content is subject to several possible sources of uncertainty. We consider the effects of four primary assumptions including model choice, uncertainty in topographic terrain, source spectrum, and conduit temperature.

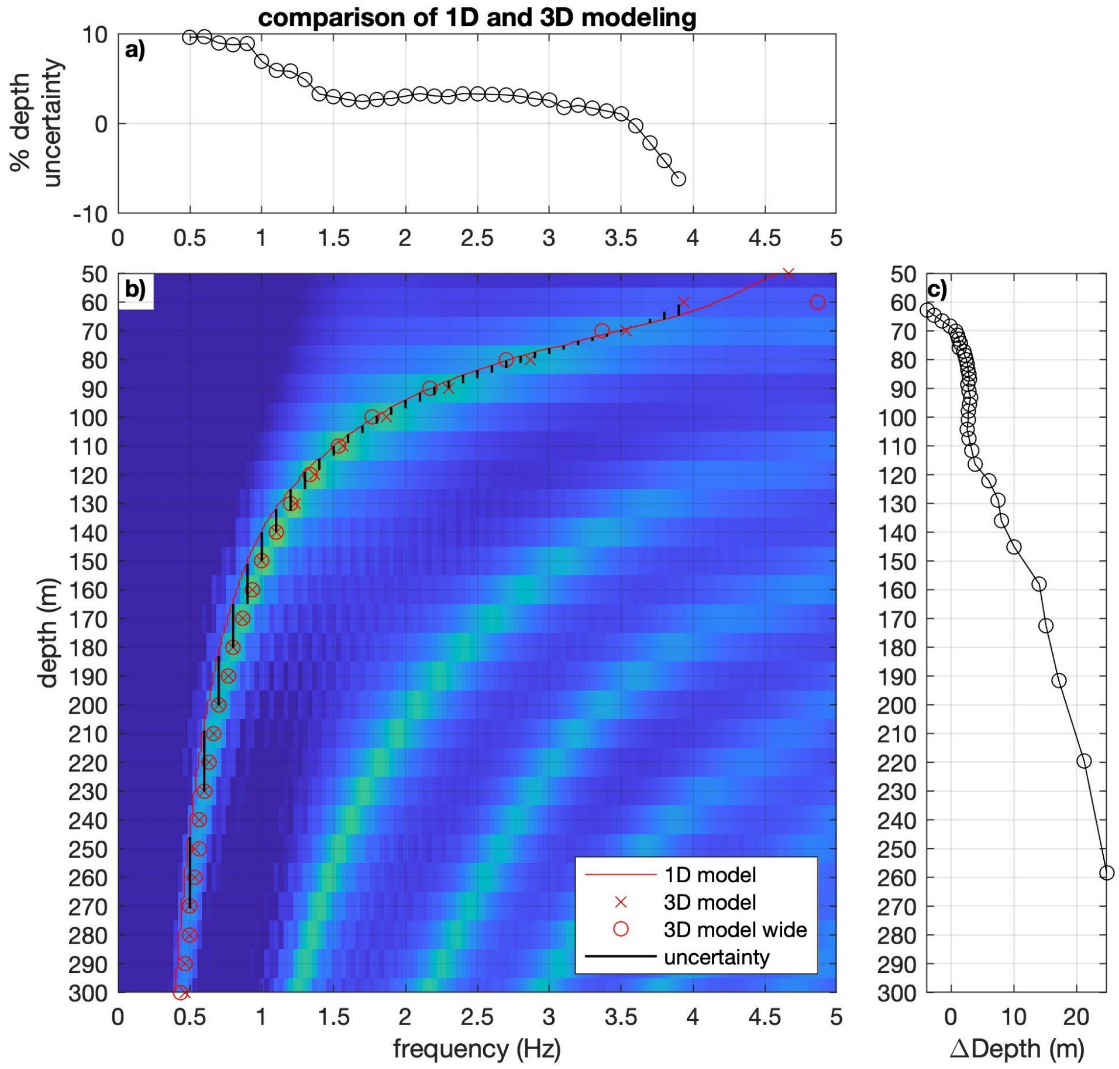

Supplementary Figure 1. *Comparison of 3D and 1D model simulations. (a) Depth (D) uncertainty as a function of frequency is calculated as 100x(D3-D1)/((D3+D1)/2) where D1 is the depth from the 1D model and D3 is the depth from the 3D model. (b) Spectrogram shows 3D model output calculated at 10 m depth increments. Peak frequency is picked from the fundamental tone and can be compared to the red line, which shows output from the CRes 1D model. Red circle symbols show peak frequency for a conduit model that has a factor of two difference in cross-sectional area compared to the profile shown in Figure 6a. (c) Difference in depth between 3D and 1D model as a function of depth, calculated by D3-D1.*

CRes 1D modeling is used in the main analysis of this paper. Here we compare it to 3D finite difference time domain modeling of acoustic wave propagation using infraFDTD (e.g., Kim & Lees, 2011). In the 3D model runs, peak frequencies are calculated as a function of magma

free surface depth using the same parameters as for 1D modeling. These include an axisymmetric SEC profile used in **Figure 6a**, a homogeneous temperature of 200 degrees C throughout SEC, and a Brune source-time function with sigma = 0.03 s. Spectrograms of the 3D output reveal that peak frequencies are in close accordance with the 1D model output (**Supplementary Figure 1**). Discrepancies of up to 25 m in the depth inversion (deeper estimates for the 3D model) are greatest at deeper depths (e.g., 250 m; error of 10%). Results imply that the 1D modeling is accurate to within 10% for all depths and to within 5% for frequencies above 1.25 Hz.

The precise topographic structure of SEC is fixed at ~10 m radius in the CRes 1D modeling, but we acknowledge this value below the crater flare is relatively poorly constrained. As such, we ran 3D models with both this conduit radius and with a larger conduit radius 1.41 times larger (which gives a cross-sectional area twice as large). Output from the wider conduit is also indicated in **Supplementary Figure 1b**, and shows negligible change in modeled frequency. We conclude that uncertainties in conduit radius do not impact depth estimates, although - as mentioned previously - they impact estimated volume fluxes.

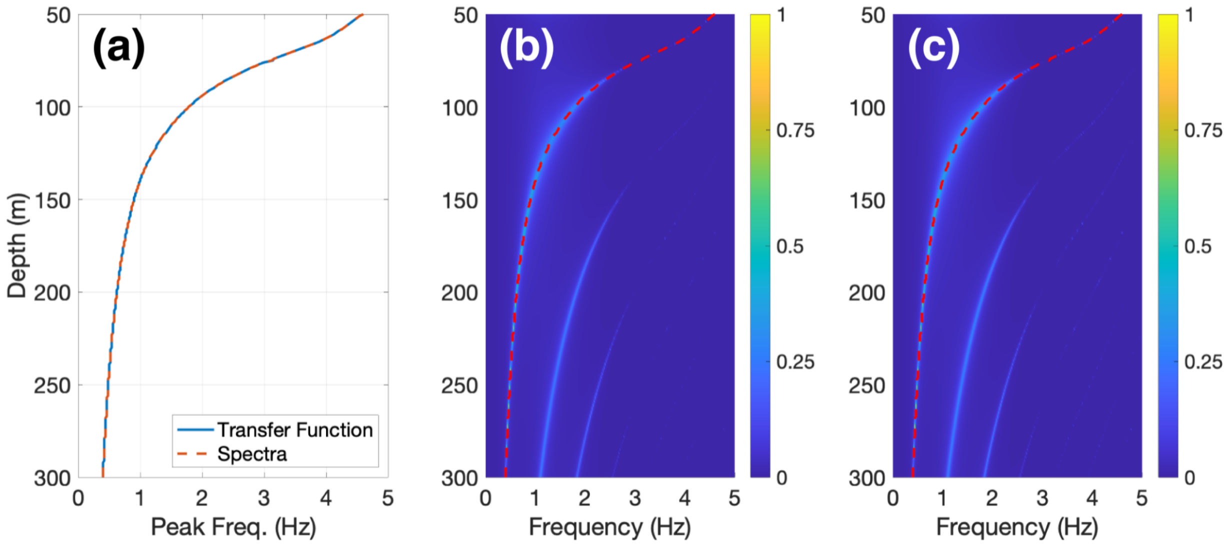

Supplementary Figure 2. *Sensitivity to source function. (a) Simulated peak frequency as a function of depth for acoustic response function (blue, solid) and spectra calculated by convolving the transfer function with a Brune source (sigma=0.03 s) (red, dashed). (b) Normalized spectrogram for spectra. (c) Normalized spectrogram for acoustic response function.*

The crater acoustic response in the CRes 1D modeling is calculated and shown in both **Figure 6b** and **Supplementary Figure 2c**, but it is important to note that the actual radiated (and recorded) infrasound will be a convolution with an arbitrary source and its own spectral content (e.g, **Supplementary Figure 2b**). Of particular note, the crater acoustic response shows prominent overtones in CRes 1D modeling, which do not appear to be obvious in the recorded gliding signals at Etna. We thus assume that the crater acoustic response function is modulated by a source dominated in low frequencies, e.g., a Brune function with sigma = 0.03 s. **Supplementary Figure 2** illustrates spectrograms calculated with both a low frequency source

function as well as an impulse. Peak frequency of the fundamental tone is consistent for both source types indicating that source function will not bias the inversion's depth estimates.

Finally, we explore varying temperature models within the conduit. Higher temperatures correspond to higher sound speeds leading to higher frequencies for a given conduit length. We use CRes 1D to test homogeneous 200 and 400 degree temperatures as well as a gradational model varying from 200 degrees to 400 degrees (**Supplementary Figure 3**). Modeling output matches expectations, which is that the error in inverted depth will be proportional to the sound speed uncertainty, e.g., percent difference in sound speed 400°C versus 200°C = sqrt(gamma*R*T = 673 K)/sqrt(gamma*R*T = 473 K) - 1 = 19%. We thus conclude that the conduit temperature uncertainty has the highest potential uncertainty for deriving magma free surface depths using infrasound. The uncertainty values quoted in the paper are due to the temperature uncertainty.

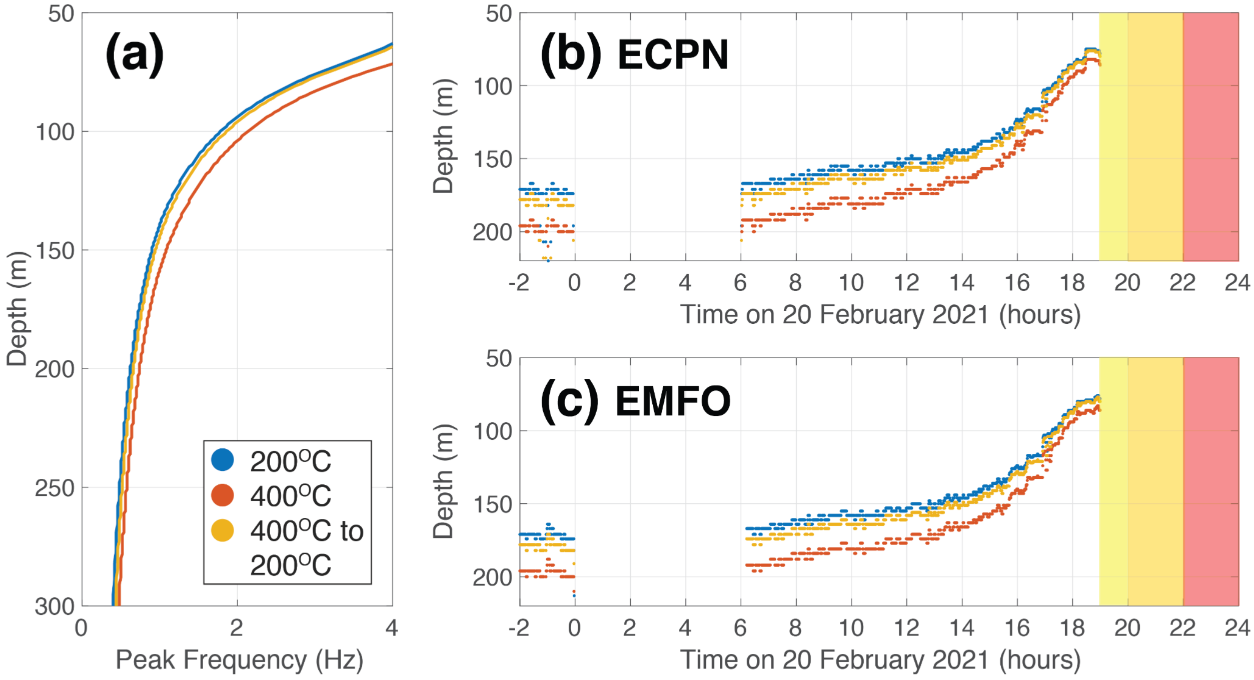

Supplementary Figure 3. *Sensitivity to temperature. (a) Simulated peak frequency as a function of depth for three temperature profiles; (blue) 200°C, (red) 400°C, and (yellow) linearly decreasing from 400°C at bottom of conduit to 200°C at top of crater. (b) and (c) Inverted depths for the three temperature profiles for station ECPN and EMFO, respectively. Colored boxes indicate different phases of the eruption: (yellow) weak Strombolian activity, (orange) strong Strombolian activity, (red) lava fountaining.*

Supplementary videos:

Video 1: *Animation Description - (top panel) 12 hours infrasound time series and spectrogram recorded at ECPN and sped up by a factor of 500 to make audible. (bottom panel) Time-synchronized thermal video shows eruptive activity from SEC including pre-eruptive infrasound gliding, Strombolian activity, and lava fountaining. Gliding-derived infrasound magma column depths are qualitatively indicated with transparent overlay beneath SEC. Depth of magma is approximate, however width of conduit is not drawn to scale.*

Video 2: *UAS (unmanned aerial system) footage recorded flying around the SEC summit area. In the foreground, the eastern side of the crater, whose rim progressively opened towards the east, is visible. Flying west, the southern and the southwestern crater rim portions, which were preserved by collapses, are shown. In the background, Bocca Nuova, Voragine, and NEC craters can be recognized respectively.*
